# Survival of adult AML patients treated with chemotherapy in the U.S. population by age, race and ethnicity, sex, calendar-year period, and AML subgroup, 2001–2019

**DOI:** 10.1016/j.eclinm.2024.102549

**Published:** 2024-03-16

**Authors:** Martha S. Linet, Rochelle E. Curtis, Sara J. Schonfeld, Jacqueline B. Vo, Lindsay M. Morton, Graça M. Dores

**Affiliations:** a Radiation Epidemiology Branch, Division of Cancer Epidemiology and Genetics, National Cancer Institute, National Institutes of Health, 9609 Medical Center Drive 7E, Rockville, MD 20850, USA; b U.S. Food and Drug Administration, Center for Drug Evaluation and Research, Office of Surveillance and Epidemiology, Silver Spring, MD, USA

**Keywords:** Acute leukemia, Myeloid leukemia, Population-based survival, Adults, Chemotherapy

## Abstract

**Background:**

Population-based survival studies of adult acute myeloid leukemia (AML) have not simultaneously evaluated age at diagnosis, race and ethnicity, sex, calendar period or AML subtypes/subgroups among chemotherapy-treated patients.

**Methods:**

For 28,473 chemotherapy-treated AML patients diagnosed at ages ≥20 years in population-based cancer registry areas of the Surveillance, Epidemiology, and End Results Program (2001–2018, followed through 2019), we evaluated 1-month through 5-year relative survival (RS) and 95% confidence intervals (95% CI) using the actuarial method in the SEER∗Stat Survival Session and overall survival (OS) using multivariable Cox regression to estimate proportional hazard ratios (HR) and 95% CI.

**Findings:**

RS decreased with increasing age (20–39, 40–59, 60–74, 75–84, ≥85 years) at AML diagnosis. RS declined substantially within the first month and, except for acute promyelocytic leukemia, decreasing patterns continued thereafter for core binding factor AML, AML with antecedent condition/therapy, and all other AML. For all ages, acute promyelocytic leukemia RS stabilized after the first year. For total AML the hazard of death was significantly increased for non-Hispanic (NH)-Black (HR = 1.18, 95% CI = 1.12–1.24) and NH-Pacific Islander patients (HR = 1.31, 95% CI = 1.11–1.55) compared with NH-White patients. In contrast, NH-Asian and Hispanic patients had similar OS to NH-White patients across all ages and most AML subgroups. Males had significantly inferior survival to females with some exceptions. Compared to 2001–2006, in 2013–2018 OS improved for all age and AML subgroups.

**Interpretation:**

Chemotherapy-treated U.S. adults with AML have notable differences in survival by age, race and ethnicity, sex, calendar-year period, and AML subgroup. Despite survival gains over time, our findings highlight the need for improving early outcomes across all AML subgroups, older ages, and Black and Pacific Islander patients and long-term outcomes among most treated groups.

**Funding:**

Intramural Research Program of the U.S. 10.13039/100000002National Institutes of Health, 10.13039/100000054National Cancer Institute, Division of Cancer Epidemiology and Genetics, and the 10.13039/100000038U.S. Food and Drug Administration, 10.13039/100022416Center for Drug Evaluation and Research, Office of Surveillance and Epidemiology.

**Research in context**

**Evidence before this study**

We searched PubMed for population-based studies through September 2023 with key terms “acute myeloid leukemia (AML) survival and adults and population-based cancer registries; ” “age at diagnosis; ” “SEER program data; ” “AML subtypes; ” “sex; ” “racial and ethnic disparities; ” and “temporal changes.” The limited number of population-based studies of AML among adults have shown large survival differences by age at diagnosis, AML subgroup, race, sex and calendar-year period. However, previous studies have not evaluated these factors simultaneously among adults of all ages while restricting the study population to individuals initially treated with chemotherapy.

**Added value of this study**

Despite recent survival gains in AML, our study showed decreasing relative survival with increasing age at AML diagnosis with a decline observed within the first month post-diagnosis for chemotherapy-treated patients in all AML subgroups. Evaluation of the impact of demographic factors and AML subgroups among chemotherapy-treated AML patients further underscored heterogeneity in overall survival by race and ethnicity, and AML clinical subgroups.

**Implications of all the available evidence**

Our results underscore the value of utilizing large population-based studies of AML in evaluating relative survival and simultaneously adjusted overall survival in adult AML patients according to specified age categories, race and ethnicity, sex, calendar-year period of diagnosis, and AML subgroups to better understand survival patterns in those receiving initial chemotherapy. Our findings highlight the need for improving early outcomes across all AML subgroups, older ages, and Black and Pacific Islander patients and long-term outcomes among most treated subgroups.

## Introduction

Adult patients with acute myeloid leukemia (AML) experience large survival differences by age at diagnosis, with substantially poorer prognosis for most patients diagnosed at ages ≥ 60 years,^1^, ^2^, ^3^, ^4^, ^5^, ^6^, ^7^, ^8^, ^9^ and by AML subtype, with the most favorable reported for acute promyelocytic leukemia (APL) and the least favorable for AML with antecedent conditions or antineoplastic therapy (AML with antecedent condition/therapy).^10^, ^11^, ^12^, ^13^ A number of population-based studies have also suggested substantial survival differences by patient subgroup, specifically, inferior survival for U.S. non-Hispanic Black patients than White patients^14^, ^15^, ^16^ and for males compared with females.^2^^,^^6^^,^^11^^,^^12^ Lastly, AML survival by calendar year period of AML diagnosis has shown modest improvements over time.^3^^,^^4^^,^^8^^,^^9^^,^^13^^,^^16^, ^17^, ^18^, ^19^ However, previous large-scale population-based analyses of AML among adults have not evaluated these factors simultaneously (*e.g.,* whether recent survival improvements are consistent across all patient subgroups) within patients initially treated with chemotherapy. AML is uniformly fatal among patients who do not receive disease-directed therapy and inclusion of these individuals together with treated patients in analyses of AML underestimates survival among treated patients.^20^

To address these gaps we report detailed findings in a population-based study of relative and overall survival among adults initially receiving chemotherapy in the Surveillance, Epidemiology, and End Results (SEER) Program over the past two decades, a period when the backbone of treatment regimens remains relevant to current clinical practice.^21^^,^^22^ In contrast to the small sample size and often strict eligibility criteria in clinical trials, our population-based study of adults treated for AML examines survival across a broad age range and diverse racial and ethnic groups, including demographic subgroups who are often underrepresented in clinical trials.^23^^,^^24^ We excluded patients not reported to SEER as initially treated with chemotherapy to prevent underestimation of potential survival gains in treated patients. This comprehensive assessment of adult AML patients treated during 2001–2018 (followed through 2019) provides a baseline by which future studies can assess survival as increasing numbers of novel AML therapies are introduced into clinical practice.^25^

## Methods

### Population

AML cases were identified from 17 population-based cancer registry areas (SEER-17) that cover 26.5% of the United States population. The SEER Program collects information on patient demographics, tumor characteristics, initial treatment in broad categories (*e.g.,* any chemotherapy, any radiotherapy), and vital status but does not include information on drug names, drug doses (neither quantitatively [specific doses] or qualitatively [*e.g.,* high-dose, low-dose, intensive, non-intensive]), subsequent treatments, performance status, or comorbid conditions. Reasons for why patients do not receive initial treatment with chemotherapy (hereafter ‘initial chemotherapy’) are not available in SEER. The term ‘initial therapy’ is defined by the SEER Program as ‘all treatments administered to the patient after the original diagnosis of cancer in an attempt to destroy or modify cancer tissue.’ Antineoplastic agents, including cytarabine, daunarubicin, arsenic trioxide (ATO), azacytidine, decitabine, and hydroxyurea are coded as chemotherapy irrespective of number of cycles, dose, or dose intensity; however, all-trans retinoic acid (ATRA), a differentiating agent, is included in SEER within the treatment category of ‘other therapy.’ Thus, patients receiving ATRA without chemotherapy are excluded from our analysis whereas patients receiving supportive treatment with hydroxyurea for control of leukocytosis without AML-directed therapy are included. SEER Program information can be found at https://seer.cancer.gov/.^26^

**Fig. 1:**
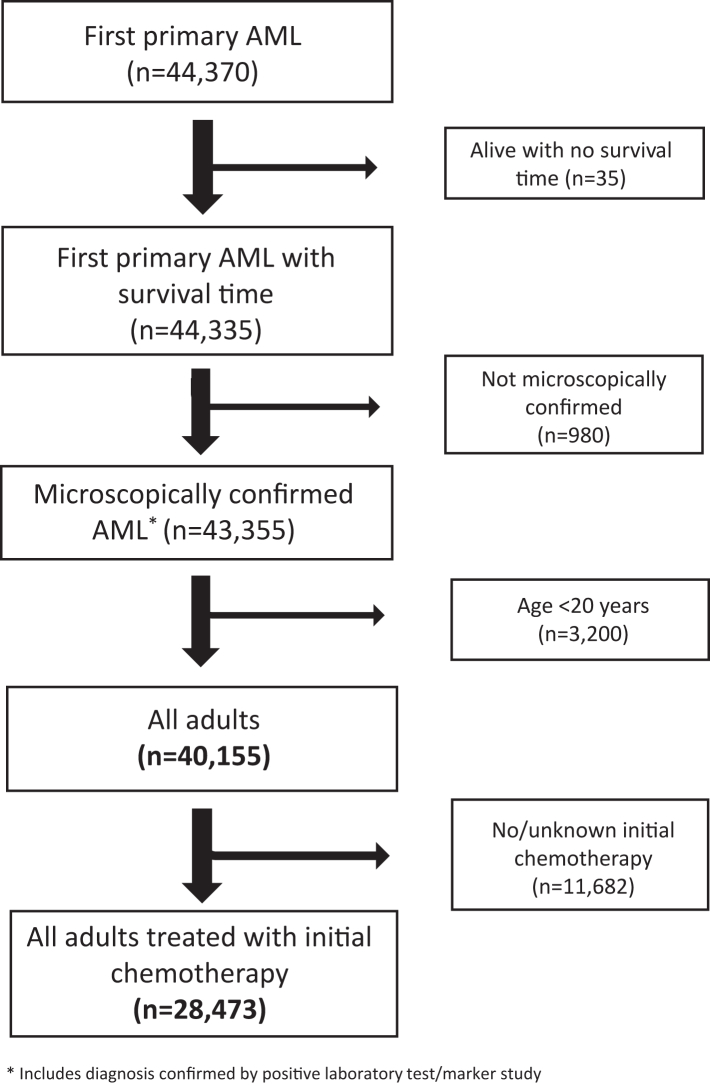
Selection of first primary acute myeloid leukemia (AML) in adults (ages ≥ 20 years at AML diagnosis) during 2001–2018 (and followed through 2019) and reported to have received chemotherapy for AML, and identified in 17 cancer registry areas of the Surveillance Epidemiology and End Results Program.

We identified 63,606 individuals with AML and excluded those diagnosed by death certificates or autopsy only (N = 602), those censored due to age exceeding expected survival tables limit of 99 years (N = 40), and those with AML occurring as a second or higher order neoplasm (N = 18,594). This resulted in 44,370 first primary AML cases diagnosed at all ages during 2001–2018 and followed through 2019. After exclusions (35 with no survival time, 980 not microscopically confirmed or diagnosed by a positive laboratory test or marker, and 3200 less than age 20, there were 40,155 confirmed cases of AML among individuals ≥20 years old at diagnosis. After excluding an additional 11,682 cases who did not receive initial chemotherapy or for whom it was unknown if they had received initial chemotherapy, the final analytic population included 28,473 adults who received initial chemotherapy (Fig. 1).

### Classification

Using the International Classification of Diseases for Oncology (third edition) (ICD-O-3) codes, we created AML subgroups based on clinical, treatment, and prognostic considerations^27^^,^^28^ and ICD-O-3 morphology codes available in SEER during the time of study.^26^ The AML subgroup categories included: acute promyelocytic leukemia (APL), core binding factor (CBF) AML, AML with antecedent condition/therapy, and all other AML (Supplementary Table S1).

### Statistical analysis

In descriptive analyses we examined the percent of adult patients known to have received initial chemotherapy for first primary AML (designated ‘total AML’) by age at diagnosis, race and ethnicity, sex, calendar period of diagnosis, and AML subtype/subgroup. Among patients treated with initial chemotherapy, we estimated relative survival (RS) defined as the ratio of observed AML survivors to expected survivors in a comparable (external) cohort from the general population matched on age, sex, race and ethnicity, and calendar year of AML diagnosis. RS and associated 95% confidence intervals (CIs) were calculated using the actuarial method in the SEER∗Stat Survival Session (version 8.4.0.1). RS was estimated at monthly and annual intervals among patients diagnosed with AML (total, by subgroup, and by subtype), overall and by age at diagnosis (20–39, 40–59, 60–74, 75–84, ≥85 years), sex, calendar year period (2001–2006, 2007–2012, 2013–2018), and race and ethnicity (Hispanic or Latino [hereafter designated Hispanic], non-Hispanic White [White], non-Hispanic Black [Black], non-Hispanic Asian or Pacific Islander [Asian or Pacific Islander], and non-Hispanic other/unknown race including American Indian [other/unknown race]). Although Asian and Pacific Islander patients are two distinct racial groups with notably different cancer burden,^29^^,^^30^ U.S. mortality data reported to the SEER Program during the study period lacked separate denominators to enable calculations of RS for each of these two racial and ethnic groups of patients. Because the numbers of AML patients ages ≥85 years at diagnosis were small in some race and ethnicity groups and in some AML subgroups, we combined the two older age groups (75–84 and ≥ 85) into a larger group (≥75) for some assessments (see below). RS estimates with <25 cases were suppressed and not shown per SEER convention. All other data points for <10 cases were suppressed to protect patient confidentiality.

For internal comparisons of overall survival (OS) for clinical, demographic and calendar period of diagnosis subgroups of AML, the dramatic variation by age at diagnosis necessitated adjustment for age and other important factors. To calculate OS we fitted multivariable Cox proportional hazards regression models using time since AML diagnosis as the time scale to calculate hazard ratios (HRs) and their associated 95% CIs. These models were mutually adjusted for age at diagnosis (20–39, 40–59, 60–74, ≥75) sex, calendar-year period (2001–2006, 2007–2012, and 2013–2018), and race and ethnicity (Hispanic, White, Black, Asian, Pacific Islander, other/unknown race). We considered 2001–2006 to be the ‘pre-hypomethylating agents (HMAs)’ treatment period and considered two subsequent calendar-year periods of equal length and follow-up (2007–2012, 2013–2018). Cox models were fitted for each of the specific age groups of AML patients evaluated that excluded all other ages. In sensitivity analyses we repeated analyses of calendar-year periods restricting follow-up time to 5 years to ensure that differences in follow-up time between the three periods did not distort the results. Descriptive statistics and Cox proportional hazard models were generated using SAS (version 9.4, Cary, NC). Two-sided P-values less than 0.05 were considered statistically significant.

### Ethics statement

This analysis used only de-identified data collected by the SEER Program and obtained through a Data Use Agreement. The study was not considered human subjects research and therefore did not require review by an Institutional Review Board.

### Role of the funding source

The study sponsor had no role in the study design; in the collection, analysis or interpretation of data; in the writing of the report; or in the decision to submit the paper for publication but did review the paper prior to submission for publication.

## Results

### Descriptive characteristics

Among 40,155 adults with first primary AML diagnosed in SEER-17 during 2001–2018, 70.9% (N = 28,473) were reported to have received initial chemotherapy (Fig. 1; Table 1). The proportion initially treated varied dramatically by age, ranging from 92.2% (ages 20–39) to 23.9% (ages ≥85). Across race and ethnicity groups, 67.1%–77.3% were reported as initially treated with chemotherapy. AML patients receiving initial chemotherapy increased substantially from 64.4% during 2001–2006 to 76.5% during 2013–2018. A slightly higher proportion of males than females were reported to receive initial chemotherapy.

**Table 1:** Frequency of adults (ages ≥ 20 years at AML diagnosis) with diagnostically confirmed first primary AML diagnosed 2001–2018 (followed through 2019) overall and for those reported to have received initial chemotherapy for AML, according to age, race and ethnicity, sex, calendar year period of diagnosis, and AML subgroups, SEER-17.^a^

Characteristic	All adults with AML (N = 40,155)	% adults treated with initial chemotherapy (70.9%)	Adults treated with initial chemotherapy (N = 28,473)									
			All adults treated with initial chemotherapy	Age (years)					AML subgroup			
				20–39	40–59	60–74	75–84	≥85	APL	CBF AML	Antecedent C/T	All other AML
	No.	%	N (%)	N (%)	N (%)	N (%)	N (%)	N (%)	N (%)	N (%)	N (%)	N (%)
Age at diagnosis, years												
20–39	4978	92.2	4592 (16.1)	–	–	–	–	–	1086 (34.8)	347 (32.0)	111 (5.6)	3048 (13.7)
40–59	10,407	88.9	9253 (32.5)	–	–	–	–	–	1298 (41.6)	407 (37.5)	496 (25.0)	7052 (31.7)
60–74	12,781	76.7	9798 (34.4)	–	–	–	–	–	602 (19.3)	253 (23.3)	888 (44.8)	8055 (36.2)
75–84	8428	47.2	3978 (14.0)	–	–	–	–	–	131 (4.2)	66 (6.1)	407 (20.5)	3374 (15.2)
≥85	3561	23.9	852 (3.0)	–	–	–	–	–	24 (0.8)	12 (1.1)	81 (4.1)	735 (3.3)
Race and ethnicity												
Non-Hispanic												
White	27,649	68.9	19,038 (66.9)	2243 (48.8)	5825 (63.0)	7245 (73.9)	3038 (76.4)	687 (80.6)	1740 (55.4)	660 (60.8)	1458 (73.5)	15,180 (68.2)
Black	3283	73.6	2417 (8.5)	533 (11.6)	941 (10.2)	692 (7.1)	211 (5.3)	40 (4.7)	343 (10.9)	92 (8.5)	124 (6.3)	1858 (8.3)
Asian	3234	75.0	2425 (8.5)	460 (10.0)	818 (8.8)	742 (7.6)	337 (8.5)	68 (8.0)	247 (7.9)	99 (9.1)	176 (8.9)	1903 (8.5)
Pacific Islander	265	74.0	196 (0.7)	44 (1.0)	84 (0.9)	53 (0.5)	15 (0.4)	0 (0)	28 (0.9)	13 (1.2)	13 (0.7)	142 (0.6)
Other/unspecified^b^	298	67.1	200 (0.7)	43 (0.9)	72 (0.8)	62 (0.6)	17 (0.4)	<10 (−)	31 (1.0)	23 (2.1)	10 (0.5)	136 (0.6)
Hispanic	5426	77.3	4197 (14.7)	1269 (27.6)	1513 (16.4)	1004 (10.2)	360 (9.0)	51 (6.0)	752 (23.9)	198 (18.2)	202 (10.2)	3045 (13.7)
Sex												
Males	21,853	72.1	15,751 (55.3)	2301 (50.1)	5034 (54.4)	5708 (58.3)	2301 (57.8)	407 (47.8)	1611 (51.3)	624 (57.5)	1239 (62.5)	12,277 (55.1)
Females	18,302	69.5	12,722 (44.7)	2291 (49.9)	4219 (45.6)	4090 (41.7)	1677 (42.2)	445 (52.2)	1530 (48.7)	461 (42.5)	744 (37.5)	9987 (44.9)
Calendar year of diagnosis												
2001–2006	12,826	64.4	8259 (29.0)	1362 (29.7)	2940 (31.8)	2674 (27.3)	1095 (27.5)	188 (22.1)	777 (24.7)	262 (24.1)	522 (26.3)	6698 (30.1)
2007–2012	13,305	71.3	9480 (33.3)	1592 (34.7)	3135 (33.9)	3175 (32.4)	1317 (33.1)	261 (30.6)	1158 (36.9)	373 (34.4)	677 (34.1)	7272 (32.7)
2013–2018	14,024	76.5	10,734 (37.7)	1638 (35.7)	3178 (34.3)	3949 (40.3)	1566 (39.4)	403 (47.3)	1206 (38.4)	450 (41.5)	784 (39.5)	8294 (37.3)
AML subgroup												
APL	3713	84.6	3141 (11.0)	1086 (23.6)	1298 (14.0)	602 (6.1)	131 (3.3)	24 (2.8)	–	–	–	–
CBF AML	1238	87.6	1085 (3.8)	347 (7.6)	407 (4.4)	253 (2.6)	66 (1.7)	12 (1.4)	–	–	–	–
AML with antecedent C/T	3009	65.9	1983 (7.0)	111 (2.4)	496 (5.4)	888 (9.1)	407 (10.2)	81 (9.5)	–	–	–	–
All other AML	32,195	69.2	22,264 (78.2)	3048 (66.4)	7052 (76.2)	8055 (82.2)	3374 (84.8)	735 (86.3)	–	–	–	–

AML, acute myeloid leukemia; APL, acute promyelocytic leukemia; CBF, core binding factor; C/T, condition/therapy; SEER-17, 17 cancer registry areas of the Surveillance, Epidemiology, and End Results Program; –, not applicable; <10, data with fewer than 10 cases are suppressed to protect patient confidentiality.

a The SEER-17 registry areas are estimated to cover 26.5% of the U.S. population and those included in this study were the states of Connecticut, Hawaii, Iowa, Kentucky, Louisiana, New Mexico, New Jersey, Utah, and registries in areas of California (San-Francisco Oakland, San Jose–Monterey, Greater California, Los Angeles), Georgia (Atlanta, Greater Georgia, Rural Georgia) and Washington state (Seattle-Puget Sound). Refer to 'Methods' section for definition of initial chemotherapy.

b Includes American Indian/Alaskan Native, other specified race, and unknown race.

The patient age distribution differed by AML subgroup and by race and ethnicity (Table 1). APL (11.0% of total AML), CBF AML (3.8%), and AML with antecedent condition/therapy (7.0%) combined accounted for a minority (21.8%) of treated patients. The majority of AML patients with APL and CBF were younger than age 60 at diagnosis. In contrast, only a very small fraction of patients with AML with antecedent condition/therapy were ages 20–39. There were 48.8% White, 11.6% Black, 10.0% Asian, 1.0% Pacific Islander, and 27.6% Hispanic patients in the youngest age group (20–39) compared with 80.6% White, 4.7% Black, 8.0% Asian, 0% Pacific Islander, and 6.0% Hispanic AML patients in the oldest age group (≥85). The majority of treated patients across all ages (except ≥85) and AML subgroups were male with the most marked male preponderance at ages 60–84 and for AML with antecedent condition/therapy. Of the total AML patients, a substantially higher proportion (76.5%) were treated with chemotherapy in the more recent calendar year period (2013–2018) compared with 64.4% in the earlier period (2001–2006).

### Relative survival

For all treated adults, RS for total AML declined with time since diagnosis from 89.9% at 1-month to 55.8%, 35.9%, and 31.9% at 1-year, 3-years, and 5-years, respectively (Table 2). For total AML, declines in survival since time of diagnosis differed notably by age but for each age group declines were greatest in the first year after diagnosis with 1-year RS below 50% for those ages 60–74, below 30% for those ages 75–84, and below 20% for those ages ≥85 years.

**Table 2:** Relative survival from 1 month to 5 years for adults (ages ≥20 years at AML diagnosis) with diagnostically confirmed first primary AML who were diagnosed 2001–2018 (followed through 2019) and reported to have received initial chemotherapy for AML according to age at diagnosis, race and ethnicity, sex, calendar year period of diagnosis, and AML subgroup/subtype, SEER-17.

Characteristic	N	Median age		Relative survival (%) according to time since AML diagnosis							
				1-month		1-year		3-year		5-year	
		Years	(IQR)	%	(95% CI)	%	(95% CI)	%	(95% CI)	%	(95% CI)
Total	28,473	60	(47–71)	89.9	(89.5–90.2)	55.8	(55.2–56.4)	35.9	(35.3–36.5)	31.9	(31.3–32.5)
Age at diagnosis, years											
20–39	4592	31	(25–35)	96.1	(95.5–96.6)	80.9	(79.7–82.0)	63.3	(61.9–64.8)	59.1	(57.6–60.6)
40–59	9253	51	(46–56)	93.3	(92.8–93.8)	66.3	(65.4–67.3)	46.9	(45.8–47.9)	42.6	(41.5–43.6)
60–74	9798	67	(63–70)	88.3	(87.7–89.0)	47.8	(46.8–48.8)	25.6	(24.7–26.5)	21.0	(20.1–21.9)
75–84	3978	78	(76–81)	82.2	(80.9–83.4)	29.7	(28.2–31.2)	8.9	(7.9–9.9)	5.6	(4.7–6.6)
≥85	852	87	(86–89)	71.4	(68.2–74.4)	18.5	(15.8–21.4)	4.6	(3.0–6.7)	2.6	(1.2–4.8)
Race and ethnicity											
Non-Hispanic											
White	19,038	63	(51–72)	89.1	(88.6–89.5)	53.8	(53.0–54.5)	34.2	(33.5–34.9)	30.0	(29.3–30.7)
Black	2417	55	(42–66)	91.3	(90.1–92.4)	56.4	(54.3–58.4)	34.7	(32.7–36.7)	31.7	(29.7–33.7)
Asian or Pacific Islander	2621	58	(43–70)	90.8	(89.6–91.9)	57.8	(55.9–59.7)	36.9	(35.0–38.9)	32.8	(30.9–34.7)
Other specified/unspecified^a^	200	56	(42–70)	89.6	(84.5–93.2)	63.5	(56.3–69.9)	46.8	(39.2–54.0)	46.2	(38.6–53.4)
Hispanic	4197	50	(36–65)	91.9	(91.0–92.7)	63.4	(61.9–64.9)	43.6	(42.0–45.2)	39.7	(38.1–41.3)
Sex											
Males	15,751	61	(48–71)	89.3	(88.8–89.8)	54.0	(53.2–54.8)	33.5	(32.7–34.3)	29.4	(28.6–30.2)
Females	12,722	59	(45–71)	90.6	(90.0–91.1)	58.1	(57.2–58.9)	38.9	(38.0–39.8)	34.9	(34.0–35.8)
Calendar year of diagnosis											
2001–2006 (follow-up 2007)	8259	59	(45–70)	88.6	(87.9–89.3)	51.1	(50.0–52.1)	31.4	(30.3–32.5)	27.9	(26.8–29.0)
2007–2012 (follow-up 2013)	9480	60	(46–70)	90.4	(89.8–91.0)	56.6	(55.6–57.6)	36.2	(35.2–37.3)	32.2	(31.1–33.3)
2013–2018 (follow-up 2019)	10,734	62	(48–72)	90.3	(89.8–90.9)	58.9	(57.9–59.8)	39.3	(38.3–40.3)	35.3	(34.2–36.4)
AML subgroup/subtype^b^											
APL	3141	47	(35–59)	90.2	(89.1–91.2)	85.0	(83.7–86.2)	82.1	(80.6–83.5)	79.9	(78.2–81.4)
CBF AML	1085	49	(35–62)	95.3	(93.8–96.4)	76.3	(73.6–78.8)	58.7	(55.5–61.7)	54.4	(51.2–57.6)
AML t(8;21)(q22;q22)	597	50	(35–63)	96.4	(94.5–97.6)	73.3	(69.5–76.7)	52.6	(48.3–56.8)	48.0	(43.6–52.2)
AML with inv(16)(p13.1q22) or t(16;16)(p13.1;q22)	488	47	(36–60)	93.9	(91.4–95.7)	79.9	(76.0–83.3)	66.1	(61.4–70.3)	62.2	(57.3–66.7)
AML with antecedent condition/therapy	1983	67	(57–74)	92.6	(91.4–93.7)	45.6	(43.3–47.8)	19.4	(17.6–21.3)	15.5	(13.8–17.3)
AML with myelodysplasia-related changes	1848	67	(57–75)	92.3	(91.0–93.5)	44.8	(42.5–47.1)	19.1	(17.2–21.0)	15.3	(13.6–17.2)
All other AML	22,264	62	(49–72)	89.3	(88.9–89.7)	51.6	(51.0–52.3)	29.7	(29.1–30.4)	25.4	(24.8–26.0)

AML, acute myeloid leukemia; APL, acute promyelocytic leukemia; CBF, core binding factor; CI, confidence interval; IQR, interquartile range; SEER-17, 17 cancer registry areas of the Surveillance, Epidemiology, and End Results Program.

a Includes American Indian/Alaskan Native, other specified race, and unknown race.

b For complete list of AML subtypes please refer to Supplementary Table S1.

**Fig. 2:**
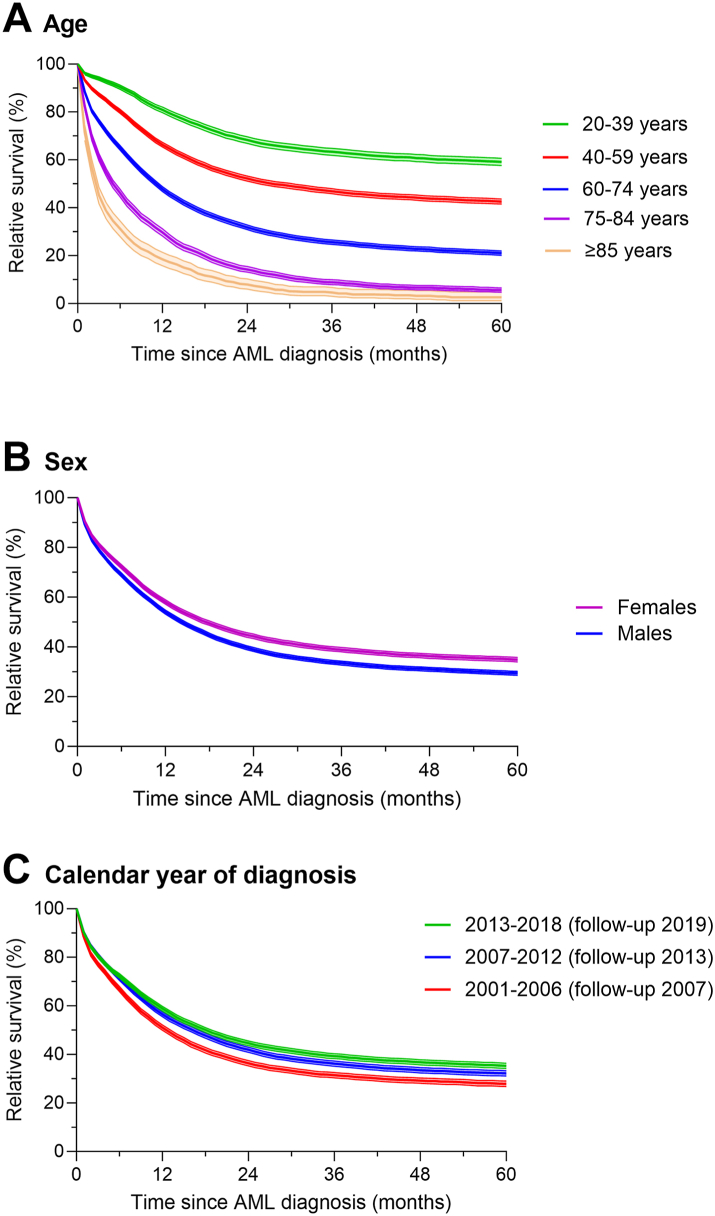
Five-year relative survival and 95% confidence intervals of adults (ages ≥ 20 years at acute myeloid leukemia (AML) diagnosis) with diagnostically confirmed first primary AML who were diagnosed 2001–2018 (followed through 2019) and reported to have received initial chemotherapy for total AML according to A) age at diagnosis, B) sex, and C) calendar year period of diagnosis identified in 17 cancer registry areas of the Surveillance, Epidemiology, and End Results Program.

**Fig. 3:**
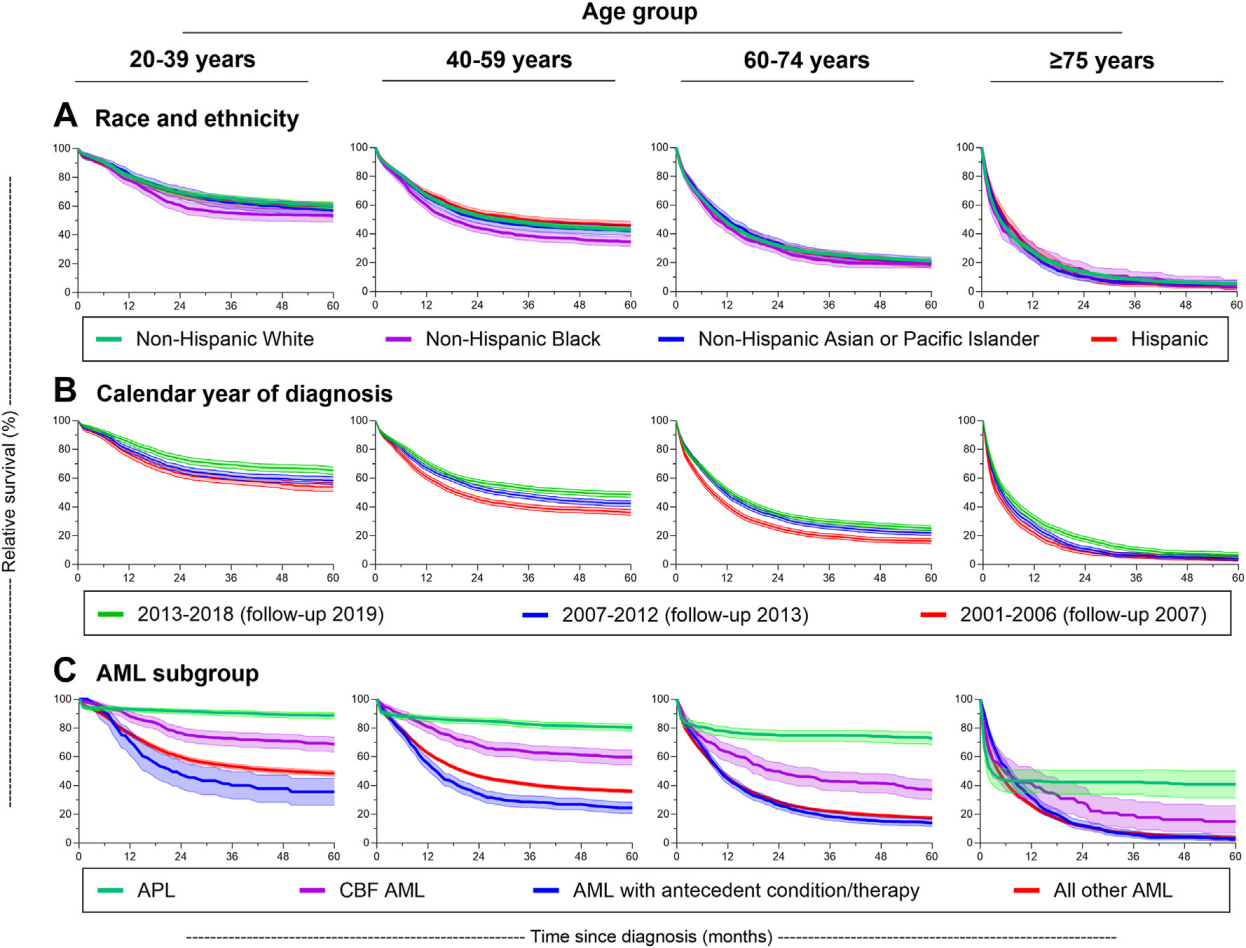
Five-year relative survival and 95% confidence intervals of adults (ages ≥ 20 years at acute myeloid leukemia (AML) diagnosis) with diagnostically confirmed first primary AML who were diagnosed 2001–2018 (followed through 2019) and reported to have received initial chemotherapy by age group according to A) race and ethnicity, B) calendar year of diagnosis, and C) AML subgroup as identified in 17 cancer registry areas of the Surveillance, Epidemiology, and End Results Program.

For total AML (Fig. 2, Table 3) and for each of the AML subgroups (Table 3, Fig. 3), notable RS differences by age at diagnosis were seen with little overlap in confidence intervals by age. Substantial reduction in RS began shortly after diagnosis with variability in the 1-month survival by age at diagnosis and AML subgroup and to a lesser extent by race/ethnicity and sex. For total AML and all AML subgroups except APL, the notable decline in RS continued during the first 2 years after diagnosis followed by a slower rate of decline for those <75 years at diagnosis in all AML subgroups (except APL). At 2-years, RS for total AML ranged from 68.3% for those diagnosed at ages 20–39 to 8.0% for those ≥85 years at diagnosis. Even for APL, there was a substantial difference in the 2-year RS by age (*e.g.,* 91.6% for those diagnosed at ages 20–39 vs. 42.3% for ages ≥ 75 years). The rate of decline in RS abated for APL patients within one year after diagnosis, most notably among the older age groups and less so among younger age groups given the latter’s excellent survival.

**Table 3:** Relative survival from 1 month to 5 years for adults (ages ≥20 years at AML diagnosis) with diagnostically confirmed first primary AML who were diagnosed 2001–2018 (followed through 2019) and reported to have received initial chemotherapy for total AML and for AML subgroups according to age at diagnosis, SEER-17.

Subgroup	No. at risk	Relative survival (%) according to time since AML diagnosis											
		1-month		1-year		2-year		3-year		4-year		5-year	
Age at diagnosis (years)		%	(95% CI)	%	(95% CI)	%	(95% CI)	%	(95% CI)	%	(95% CI)	%	(95% CI)
Total AML													
20–39	4592	96.1	(95.5–96.6)	80.9	(79.7–82.0)	68.3	(66.9–69.7)	63.4	(61.9–64.8)	60.7	(59.2–62.2)	59.1	(57.6–60.6)
40–59	9253	93.3	(92.8–93.8)	66.3	(65.4–67.3)	52.2	(51.1–53.2)	46.9	(45.8–47.9)	44.2	(43.1–45.3)	42.6	(41.5–43.6)
60–74	9798	88.3	(87.7–89.0)	47.8	(46.8–48.8)	31.8	(30.9–32.8)	25.6	(24.7–26.5)	22.8	(21.9–23.7)	21.0	(20.1–21.9)
75–84	3978	82.2	(80.9–83.4)	29.7	(28.2–31.2)	14.2	(13.1–15.4)	8.9	(7.9–9.9)	6.6	(5.7–7.6)	5.6	(4.7–6.6)
≥85	852	71.4	(68.2–74.4)	18.5	(15.8–21.4)	8.0	(6.0–10.3)	4.6	(3.0–6.7)	3.2	(1.8–5.4)	2.6	(1.2–4.8)
APL													
20–39	1086	94.6	(93.1–95.8)	93.0	(91.3–94.4)	91.6	(89.8–93.2)	90.4	(88.5–92.1)	89.4	(87.3–91.2)	88.7	(86.5–90.6)
40–59	1298	90.9	(89.2–92.4)	86.8	(84.8–88.5)	85.0	(82.9–86.9)	82.7	(80.4–84.8)	81.4	(79.0–83.6)	80.4	(77.9–82.7)
60–74	602	86.7	(83.7–89.1)	77.5	(73.8–80.7)	74.9	(71.0–78.4)	74.9	(71.0–78.4)	74.2	(69.9–77.9)	72.6	(68.1–76.6)
≥75	155	66.1	(58.0–73.0)	43.3	(35.2–51.1)	42.3	(34.0–50.5)	42.3	(34.0–50.5)	40.9	(31.4–50.2)	40.9	(31.4–50.2)
CBF AML													
20–39	347	98.6	(96.6–99.4)	88.1	(84.2–91.1)	76.6	(71.7–80.8)	72.9	(67.7–77.4)	70.9	(65.6–75.6)	68.7	(63.2–73.5)
40–59	407	96.8	(94.6–98.2)	80.9	(76.6–84.4)	68.0	(63.1–72.4)	63.3	(58.3–68.0)	61.2	(56.0–66.0)	59.8	(54.5–64.7)
60–74	253	93.0	(89.1–95.6)	63.3	(56.9–69.1)	49.9	(43.3–56.1)	43.0	(36.4–49.5)	41.5	(34.8–48.1)	36.8	(30.0–43.7)
75+	78	79.9	(69.0–87.3)	41.4	(29.9–52.5)	28.2	(18.0–39.3)	19.5	(10.7–30.1)	16.3	(8.0–27.1)	14.9	(6.9–25.9)
AML with antecedent condition/therapy													
20–39	111	100	–	70.7	(61.2–78.3)	47.6	(37.8–56.8)	40.2	(30.6–49.5)	37.9	(28.4–47.3)	35.6	(26.3–45.0)
40–59	496	94.8	(92.4–96.4)	55.3	(50.8–59.6)	34.3	(30.0–38.5)	28.7	(24.7–32.9)	27.0	(23.0–31.1)	24.5	(20.6–28.5)
60–74	888	92.3	(90.3–93.9)	44.5	(41.2–47.8)	26.3	(23.3–29.4)	18.5	(15.8–21.3)	15.1	(12.6–17.8)	14.0	(11.5–16.7)
≥75	488	89.4	(86.2–91.9)	31.6	(27.4–36.0)	12.4	(9.4–15.7)	5.7	(3.6–8.4)	4.0	(2.3–6.6)	2.6	(1.1–5.2)
All other AML													
20–39	3048	96.2	(95.5–96.9)	76.1	(74.6–77.6)	59.8	(58.0–61.6)	53.4	(51.6–55.3)	50.2	(48.3–52.0)	48.3	(46.5–50.2)
40–59	7052	93.4	(92.8–94.0)	62.5	(61.4–63.7)	46.5	(45.4–47.7)	40.7	(39.5–41.9)	37.6	(36.5–38.8)	36.0	(34.8–37.2)
60–74	8055	87.9	(87.2–88.6)	45.4	(44.3–46.5)	28.7	(27.6–29.7)	22.1	(21.1–23.1)	19.2	(18.3–20.2)	17.4	(16.5–18.4)
≥75	4109	79.8	(78.5–81.0)	26.4	(25.0–27.8)	11.8	(10.8–12.9)	6.8	(6.0–7.8)	4.7	(3.9–5.5)	3.7	(3.0–4.6)

AML, acute myeloid leukemia; APL, acute promyelocytic leukemia; CBF, core binding factor; CI, confidence interval; RS, relative survival; SEER-17, 17 cancer registry areas of the Surveillance, Epidemiology, and End Results Program; –, not applicable.

RS was more favorable in females than males and was substantially improved in 2013–2018 compared with 2001–2006, with a few exceptions (Table 2, Fig. 2). Adult patients treated for APL experienced the most favorable RS demonstrating stable levels within the first year after diagnosis at all ages, even at ages ≥75 years (Table 3, Fig. 3). Patients treated for AML with antecedent condition/therapy had the least favorable RS of all AML subgroups; among those ages ≥75 years, RS at 3 years was 5.7%. Patients with ‘total AML’ of all ages and in each age group experienced improved relative survival in 2013–2018 compared with 2001–2006.

### Overall survival (OS)

To adjust for substantial differences in age at AML diagnosis when comparing survival across sex, race and ethnicity, calendar year of diagnosis, and other subgroups we evaluated multivariable Cox proportional hazards models for OS. As expected, compared to individuals ages 20–39, HRs rose dramatically with increasing age for treated patients with total AML and all subgroups (Table 4). Compared with White patients, the HRs for total AML were significantly increased for Black patients (HR = 1.18, 95% CI = 1.12–1.24, N = 2417) and for Pacific Islander patients (HR = 1.31, 95% CI = 1.11–1.55, N = 196). Adjusted hazard ratios among Black patients ranged from 1.16 (95% CI = 0.93–1.46) (APL) to 1.33 (95% CI = 0.97–1.81) (CBF AML) and among Pacific Islander patients from 1.16 (95% CI = 0.52–2.59) (APL) to 1.85 (95% CI = 1.07–3.20) (AML with antecedent condition/therapy, based on only 13 cases). Adjusted OS was remarkably similar for total AML among White, Asian, and Hispanic patients with HRs for Asian and Hispanic patients not differing from White patients within AML subgroups. Across AML subgroups, hazard ratios were largely lower for females (vs. males) ranging from 0.89 to 0.98. For total AML and almost all AML subgroups except AML with antecedent condition/therapy, the hazard ratios for OS were significantly lower in 2013–2018 compared to 2001–2006. This finding was confirmed in analyses that limited patient follow-up to 5-years in both calendar periods (data not shown).

**Table 4:** Multivariable Cox proportional hazards analysis for adults (ages ≥20 years at AML diagnosis) with diagnostically confirmed first primary AML who were diagnosed 2001–2018 (followed through 2019) and reported to have received initial chemotherapy for AML comparing adjusted risks for overall survival for total AML and AML subgroups according to age at diagnosis, race and ethnicity, sex, and calendar year period of diagnosis, SEER-17.

Characteristic	Total AML			APL			CBF AML			AML with antecedent condition/therapy			All other AML		
	No. at risk	HR (95% CI)	P value	No. at risk	HR (95% CI)	P value	No. at risk	HR (95% CI)	P value	No. at risk	HR (95% CI)	P value	No. at risk	HR (95% CI)	P value
Total	28,473	–		3141	–		1085	–		1983	–		22,264	–	
Age at diagnosis, years															
20–39	4592	1.00		1086	1.00		347	1.00		111	1.00		3048	1.00	
40–59	9253	1.72 (1.63–1.81)	<0.0001	1298	1.98 (1.62–2.42)	<0.0001	407	1.47 (1.16–1.86)	0.002	496	1.54 (1.19–2.00)	0.001	7052	1.52 (1.44–1.61)	<0.0001
60–74	9798	3.30 (3.13–3.47)	<0.0001	602	3.75 (3.03–4.65)	<0.0001	253	3.03 (2.37–3.86)	<0.0001	888	2.24 (1.74–2.88)	<0.0001	8055	2.75 (2.60–2.91)	<0.0001
≥75	4830	6.11 (5.78–6.46)	<0.0001	155	10.50 (8.14–13.56)	<0.0001	78	6.43 (4.73–8.75)	<0.0001	488	3.55 (2.74–4.59)	<0.0001	4109	4.97 (4.68–5.28)	<0.0001
Race and ethnicity															
Non-Hispanic															
White	19,038	1.00		1740	1.00		660	1.00		1458	1.00		15,180	1.00	
Black	2417	1.18 (1.12–1.24)	<0.0001	343	1.16 (0.93–1.46)	0.19	92	1.33 (0.97–1.81)	0.07	124	1.21 (0.99–1.48)	0.06	1858	1.23 (1.16–1.30)	<0.0001
Asian	2425	0.98 (0.94–1.04)	0.54	247	0.89 (0.67–1.17)	0.40	99	0.78 (0.57–1.08)	0.13	176	0.94 (0.79–1.12)	0.49	1903	1.01 (0.95–1.06)	0.87
Pacific Islander	196	1.31 (1.11–1.55)	0.002	28	1.16 (0.52–2.59)	0.73	13	1.52 (0.67–3.42)	0.31	13	1.85 (1.07–3.20)	0.03	142	1.38 (1.14–1.66)	0.0007
Other/unspecified^a^	200	0.90 (0.75–1.09)	0.29	31	0.63 (0.26–1.52)	0.30	23	0.96 (0.51–1.81)	0.90	10	0.95 (0.49–1.84)	0.88	136	1.05 (0.85–1.30)	0.64
Hispanic	4197	1.00 (0.96–1.04)	0.88	752	1.11 (0.93–1.34)	0.26	198	1.09 (0.86–1.37)	0.48	202	1.03 (0.87–1.22)	0.73	3045	1.05 (1.00–1.10)	0.05
Sex															
Males	15,751	1.00		1611	1.00		624	1.00		1239	1.00		12,277	1.00	
Females	12,722	0.89 (0.87–0.92)	<0.0001	1530	0.91 (0.79–1.04)	0.17	461	0.98 (0.82–1.16)	0.79	744	0.94 (0.85–1.03)	0.19	9987	0.89 (0.86–0.92)	<0.0001
Calendar year of diagnosis															
2001–2006	8259	1.00		777	1.00		262	1.00		522	1.00		6698	1.00	
2007–2012	9480	0.85 (0.82–0.88)	<0.0001	1158	0.80 (0.67–0.94)	0.006	373	0.95 (0.77–1.17)	0.61	677	0.91 (0.80–1.02)	0.11	7272	0.87 (0.84–0.91)	<0.0001
2013–2018	10,734	0.74 (0.72–0.77)	<0.0001	1206	0.60 (0.49–0.72)	<0.0001	450	0.74 (0.59–0.92)	0.008	784	0.91 (0.81–1.03)	0.14	8294	0.75 (0.72–0.78)	<0.0001

AML, acute myeloid leukemia; APL, acute promyelocytic leukemia; CBF, core binding factor; CI, confidence interval; HR, hazard ratio; SEER-17, 17 cancer registry areas of the Surveillance, Epidemiology, and End Results Program; –, not applicable.

a Includes American Indian/Alaskan Native, other specified race, and unknown race.

For all treated adults with AML, adjusted risks for OS within age categories revealed significantly increased HRs for Black (vs. White) patients in all age groups under age 75 years and for Pacific Islander (vs. White) patients in age groups 40–74 years (Table 5). Significantly reduced hazard ratios (HRs ranging from 0.82 to 0.90) were observed for female patients (vs. males), except in the oldest age group. Hazard ratios for OS were significantly lower (HRs ranged from 0.68 to 0.76) for patients diagnosed in 2013–2018 (vs. 2001–2006) across all four age groups.

**Table 5:** Multivariable Cox proportional hazards analysis for adults (ages ≥20 years at AML diagnosis) with diagnostically confirmed first primary AML who were diagnosed 2001–2018 (followed through 2019) and reported to have received initial chemotherapy for AML comparing adjusted risks for overall survival of total AML for age at diagnosis subgroups according to race and ethnicity, sex, and calendar year period of diagnosis, SEER-17.

Characteristic	Age at diagnosis (years)											
	20–39			40–59			60–74			≥75		
	No. at risk	HR (95% CI)	P value	No. at risk	HR (95% CI)	P value	No. at risk	HR (95% CI)	P value	No. at risk	HR (95% CI)	P value
Total	4592	–		9253	–		9798	–		4830	–	
Race and ethnicity												
Non-Hispanic												
White	2243	1.00		5825	1.00		7245	1.00		3725	1.00	
Black	533	1.31 (1.14–1.50)	0.0002	941	1.28 (1.17–1.39)	<0.0001	692	1.10 (1.01–1.20)	0.03	251	1.05 (0.92–1.20)	0.49
Asian	460	1.04 (0.89–1.21)	0.63	818	1.00 (0.91–1.10)	0.94	742	0.95 (0.87–1.03)	0.21	405	1.00 (0.90–1.12)	0.96
Pacific Islander	44	1.00 (0.62–1.59)	0.98	84	1.39 (1.08–1.80)	0.01	53	1.35 (1.01–1.81)	0.05	15	1.50 (0.90–2.48)	0.12
Other/unspecified^a^	43	0.70 (0.40–1.24)	0.23	72	0.76 (0.54–1.06)	0.10	62	1.11 (0.83–1.49)	0.48	23	1.02 (0.64–1.63)	0.92
Hispanic	1269	1.02 (0.92–1.14)	0.71	1513	0.96 (0.89–1.04)	0.34	1004	1.04 (0.96–1.12)	0.35	411	0.98 (0.88–1.09)	0.68
Sex												
Males	2301	1.00		5034	1.00		5708	1.00		2708	1.00	
Females	2291	0.82 (0.75–0.90)	<0.0001	4219	0.90 (0.85–0.95)	<0.0001	4090	0.86 (0.82–0.90)	<0.0001	2122	0.98 (0.93–1.04)	0.54
Calendar year of diagnosis												
2001–2006	1362	1.00		2940	1.00		2674	1.00		1283	1.00	
2007–2012	1592	0.88 (0.79–0.98)	0.02	3135	0.84 (0.79–0.89)	<0.0001	3175	0.83 (0.79–0.88)	<0.0001	1578	0.88 (0.82–0.95)	0.001
2013–2018	1638	0.68 (0.61–0.77)	<0.0001	3178	0.72 (0.68–0.77)	<0.0001	3949	0.76 (0.72–0.81)	<0.0001	1969	0.76 (0.71–0.82)	<0.0001

AML, acute myeloid leukemia; CI, confidence interval; HR, hazard ratio; SEER-17, 17 cancer registry areas of the Surveillance, Epidemiology, and End Results Program; –, not applicable.

a Includes American Indian/Alaskan Native, other specified race, and unknown race.

## Discussion

Our large comprehensive study is one of few U.S. population-based investigations of AML in adults to examine RS at multiple time points since diagnosis as well as the impact of age at diagnosis, race and ethnicity, sex, calendar period, and AML subgroups on multivariable adjusted OS (hazard ratios) for patients diagnosed at all adult ages and initially treated with chemotherapy. For chemotherapy-treated patients in all AML subgroups, survival decreased with increasing age at diagnosis with a notable death rate within the first month after diagnosis. Declines in RS during the first two years after diagnosis were also substantial across all age and AML subgroups, except for APL where RS was uniformly excellent among the younger age groups and survival declines abated within one year after diagnosis among the older age groups. In the most detailed analysis of AML survival differences among U.S. race and ethnic groups to date, the adjusted HRs for total AML were significantly increased for Black and Pacific Islander patients compared to White patients, for Black patients compared with White patients for all age groups less than 75 years and for Pacific Islander patients at ages 40–74 but were generally similar among Asian and Hispanic patients compared with White patients across all AML subgroups. HRs were lower for females than males across all age groups except the oldest age group, and for total AML. This pattern of more favorable survival for females compared with males was seen across all AML subgroups with HRs not reaching significance for patients with APL, CBF AML, and AML with antecedent condition/therapy. Lastly, adult patients of all ages treated with chemotherapy experienced significantly improved survival during 2013–2018 in most AML subgroups, except those with AML with antecedent condition/therapy, compared with those diagnosed in 2001–2006. These results demonstrate the wide heterogeneity in survival outcomes among treated AML patients and despite survival gains over time, highlight patient subgroups and timeframes post-diagnosis that can be targeted more urgently for improvement.

Much of the survival information for adults treated with chemotherapy for AML has been gleaned from clinical trials, with few population-based studies considering patient treatment status. For this reason, we are limited in comparisons that can be made with other population-based studies because few were restricted to adult patients of a broad age range initially treated with chemotherapy, and most did not compare survival among specific AML subgroups/subtypes, narrower more homogeneous age groups or many racial and ethnic groups.^3^^,^^4^^,^^9^^,^^14^, ^15^, ^16^^,^^18^^,^^19^^,^^31^ Three population-based studies of SEER linked with Medicare have examined AML survival in chemotherapy-treated patients but focused exclusively on individuals older than 65 years,^2^^,^^7^^,^^8^ limiting comparisons with younger patients. A small number of other studies assessed AML survival in populations treated with chemotherapy. These included a Danish population-based study conducted during 2002–2016 of 3820 adult AML patients among whom the 1867 ‘fit’ for intensive treatment demonstrated significantly improved 2-year OS (that remained significant after adjusting for age) compared with those receiving non-intensive, palliative or no treatment.^31^ A summary of U.K. clinical trials over 25 years (1988–2014) revealed meaningful improvements in survival for adults <60 years treated with intensive chemotherapy but less so for those ≥60 years over the time period studied.^32^ In addition, a U.S. population-based study of patients treated for APL reported worse one-month mortality and OS associated with greater co-morbidity burden in adults ≥60 years than in younger patients,^33^ and an APL study from Hong Kong described the proportion of early deaths decreasing from 28% in the 1990s to 15% during the past two decades.^34^ Our finding that a disproportionately small fraction of older adults were reported to have been treated with chemotherapy (47.2% of those 75–84 and 23.9% of those ages ≥85 years at diagnosis) highlights the importance of considering treatment status and age when assessing survival estimates over time.

Chemotherapy-treated adult AML patients of all ages and in most AML subgroups experienced substantially improved RS in 2013–2018 compared with 2001–2006 except for those with AML with antecedent condition/therapy. Particularly notable was the remarkable improvement over time for adult patients treated for APL and the stable survival estimates for APL among all age groups who survived the initial year after diagnosis. During the 18-year period of our study, cytarabine and anthracycline combinations (*e.g.,* “7 + 3”) remained the cornerstone of non-APL AML treatment, with novel agents beginning to emerge in 2017.^9^ The HMAs azacitidine and decitabine were approved by the U.S. Food and Drug Administration in 2004 and 2006, respectively, and a SEER-Medicare study of older adults with AML showed increasing use of low intensity HMA-based treatment for AML patients diagnosed during 2000–2009, ranging from 13.6% of those ages 66–70 to 36.8% of those >80 years.^8^ The availability of HMAs may account for our observation of a substantially higher proportion of individuals 60–74, 75–84, and ≥85 years treated with chemotherapy in 2013–2018 compared to 2001–2006. With few novel agents introduced during our study period, the significantly lower hazard ratios among AML patients diagnosed in the most recent calendar period across nearly all AML subgroups and age groups suggest that improvements in supportive care and treatment approaches (*e.g.,* transplantation), likely contributed to this survival advantage. Other population-based U.S.^3^^,^^9^^,^^13^^,^^16^^,^^18^ and European^4^^,^^17^^,^^19^ studies reported generally modest improvements in survival over time, but these studies combined chemotherapy-treated and untreated AML patients, and few assessed temporal changes for multiple AML subgroups and/or narrower groupings by age at diagnosis.

The induction of remission of APL following FDA approval of ATRA in 1995 and ATO in 2000 resulted in dramatic improvements in survival,^35^ and the importance of prompt treatment of APL with ATRA has been underscored in clinical trials.^32^ An earlier (1992–2007) SEER-based study of APL identified higher 1-month death rates than reported in clinical trials and only modest calendar time improvement for this short period post-diagnosis, particularly among older individuals.^36^ Nevertheless, our data reveal that early deaths persist, particularly among older individuals. Early consideration of the diagnosis of APL in all age groups, prompt initiation of ATRA, and aggressive supportive care remain critical for this patient population.

For all AML subgroups we found notable differences in adjusted OS by age at diagnosis, with more narrowly defined age groups we evaluated that are typically are masked in previous studies considering only broad age groups. Clinical studies are frequently too small to provide stable age group estimates of AML survival and often not generalizable to the population at large. Even the 25-year summary of U.K. clinical trials lacked detail for narrow age categories or survival by sex.^32^ The Danish population-based study noted that use of intensive chemotherapy increased substantially in patients ages 50–75 during 2000–2016 and that significant improvements in 2-year OS were exclusively observed in patients aged ≥50 at diagnosis who were treated with intensive chemotherapy.^31^ Other U.S. population-based studies considered survival for a limited number of AML subgroups but did not focus on chemotherapy-treated patients.^13^^,^^18^^,^^37^ Although Maynadie and colleagues^10^ reported RS within 3 adult age groups for many AML subtypes in a large population-based European study, a U.K. population-based study focused on detailed AML subtypes within sex rather than age groups.^11^ Mournier et al.^12^ modelled the influence of age at diagnosis in a French population-based study, and Sasaki et al.^13^ evaluated 5-year survival for several AML subtypes for multiple age groups and calendar-year periods within SEER. None of these studies^10^, ^11^, ^12^, ^13^ restricted analyses to chemotherapy-treated patients. Survival was poor for patients older than age 65 in European studies^4^^,^^5^ and in U.S. SEER-Medicare studies,^2^^,^^3^^,^^6^^,^^7^ but the U.S. SEER-Medicare linked studies did not examine survival by AML subtype/subgroups.^2^^,^^7^^,^^8^

Our study, the first to evaluate survival differences in chemotherapy-treated AML patients by race and ethnicity according to AML subgroups within 4–5 age groups, observed significantly higher hazard ratios for chemotherapy-treated Black patients diagnosed with AML at younger ages than White patients but similar survival for Asian and Hispanic compared to White patients. The results also revealed notably worse adjusted OS for total AML among Pacific Islander patients (similar to the poorer survival of Black patients) compared to White patients. Our findings of worse OS for Pacific Islander compared to White patients, a difference not seen for Asian vs. White patients, is consistent with disparities in death rates reported in the United States for Asian vs. Pacific Islander patients for several other cancer sites.^30^ As such, RS findings for the aggregated group of Asian and Pacific Islander patients was likely driven by Asian patients who comprised 92% of the group. Our findings reinforce the need to disaggregate Asian from Pacific Islander as separate racial groups and warrant future examination of factors driving the poorer outcomes among Pacific Islander populations.^29^ Previous SEER studies evaluated differences in AML survival by racial and ethnic groups without focusing on chemotherapy-treated AML patients. Similar to our results of poorer survival for Black patients compared to White patients, Bhatnagar and colleagues^16^ found inferior survival for Black patients for total AML compared to White patients in SEER-9 (1986–2015) and speculated that Black patients were less likely to receive intensive chemotherapy or allogeneic stem cell transplantation compared with White patients.^16^ Also, these investigators hypothesized that specific mutations that may impact outcome differ by racial and ethnic group based on their data from the Cancer and Leukemia Group B/Alliance trials where they observed that OS was not improved in favorable-risk *NPM1*-mutated/*FLT3-IHD*^low/no^ AML in young Black patients compared with White patients, albeit based on small numbers of cases.^16^ In a SEER-17 analysis (1999–2008) of AML survival, Patel and colleagues^14^ described increased hazard ratios for mortality among Black (12%) and Hispanic (6%) patients compared to White patients and noted that Black and Hispanic AML patients were diagnosed at younger ages than White patients. Black patients had a slightly higher prevalence of the more favorable subtype (t(8;21)) than White patients but had a higher AML mortality than the other racial and ethnic groups.^15^ The discrepancy in findings for Hispanic AML patients between the Patel et al. study compared with our study may reflect our exclusions of patients not treated with chemotherapy, patients with second or later primary AML and pediatric patients and/or differences in study period.^14^^,^^15^ Other hypothesized reasons for the racial and ethnic survival differences include pre-treatment morbidities and performance status, structural barriers to healthcare access related to socioeconomic status (individual and area-level), other social determinants of health not measurable within SEER, cytogenetic and molecular abnormalities, intensity and type of treatment (including differences in drug metabolism), and post-treatment differences in follow-up care.^16^^,^^38^

Our investigation among chemotherapy-treated patients with total AML and subgroups found that females had notably more favorable RS than males at all time intervals assessed following diagnosis, and significantly higher overall survival at all ages except among those ≥75 years. Most population-based studies have shown a survival advantage for AML in females and similar to our findings, two SEER-Medicare population-based studies of treated patients did not find a sex difference in survival at older ages.^7^^,^^8^ Rausch and colleagues^39^ identified male sex to be associated with unfavorable risk largely due to significantly higher proportion of adverse-risk genetic mutations including higher prevalence of *RUNX1* and *ASXL1* mutations and lower prevalence of *NPM1* mutations.

Strengths of our investigation of AML survival include the population-based nature, recent time period (*e.g.,* patients diagnosed during 2001–2018), large size, and substantial coverage of racial and ethnic subgroups in the adult U.S. population. Novel aspects of our study were the focus on the broad age span of adult AML patients initially treated with chemotherapy and assessment of early and late RS for all of the adult age groups according to AML subgroup. Also new were multivariable hazard ratio comparisons of racial and ethnic groups, sex, and calendar year period of diagnosis for total AML and AML subgroups, and for total AML within the four age groups and according to race and ethnicity, sex, and calendar year period among patients treated with chemotherapy.

Limitations of our data include lack of information about specific chemotherapy agents and subsequent therapy. To this end, we could not exclude patients receiving only supportive chemotherapy (*e.g.,* hydroxyurea) without AML-directed treatment; however, inclusion of these patients would most likely result in underestimation of survival. We also could not determine reasons for patients not receiving initial chemotherapy since this information is not available in the SEER registries. Although SEER does not collect information on performance status and comorbidities, based on our selection criteria our study population was deemed to be sufficiently fit for treatment at the time of AML diagnosis. Other limitations include lack of centralized pathology review, AML disease categories limited by the available ICD-O-3 codes during the study period, and the small numbers of cases in certain AML subtypes despite the large study population. We also did not have information on prognostic molecular markers such as *p53, NPM1,* and *FLT3,* although with rare exception, this information likely would not have influenced initial treatment choice during our study period.

In summary, this is one of the first large U.S. population-based investigations to evaluate RS and adjusted OS in adult AML patients initially treated with chemotherapy within AML subgroups according to specified age categories, race and ethnicity, sex, and calendar year period. Our study highlights the importance of considering these factors when quantifying survival for AML subgroups and the potential for more comprehensive investigation of survival with inclusion of disease and treatment characteristics by patient subgroup. Future research is needed to comprehensively evaluate the underlying reasons for the race and ethnicity findings we observed. The results also point to the particular need for improving early survival outcomes across all AML subgroups, older ages, and Black, and Pacific Islander patients. The progressive decline in RS with time since diagnosis for patients with all AML subtypes, except for APL, also provides directions for future improvements in care, while the development and approval of a growing number of promising agents since 2017^40^ underscores the need to continue monitoring survival over time in population-based studies.

## Contributors

MSL, REC, SJS, JBV, LMM, and GMD designed the study and analysis. All authors had access to the data. REC and GMD conducted the formal analysis and verified the data. MSL wrote the first draft. MSL, REC, SJS, JBV, LMM, and GMD revised the paper for scientific content and edited the paper. MSL, REC, SJS, JBV, LMM, and GMD made the decision to submit for publication. All authors read and approved the final version of the manuscript.

## Data sharing statement

The data used for this study are available upon application to the SEER Program (seer.cancer.gov).

## Declaration of interests

The authors have no relationships to disclose.
