# Supplementary material for: Survival of adult AML patients treated with chemotherapy in the U.S. population by age, race and ethnicity, sex, calendar-year period, and AML subgroup, 2001–2019
Source: eClinicalMedicine. 2024 Mar 16;71:102549. doi: 10.1016/j.eclinm.2024.102549 (PMC10957373; doi:10.1016/j.eclinm.2024.102549)
Supplement: Supplementary Table S1 [file mmc1.pdf]

Table S1. Frequency distribution, International Classification of Diseases for Oncology (third edition) codes, median age, and relative survival of AML subgroups/subtypes of adults (ages ≥20 years at AML diagnosis) with diagnostically confirmed first primary AML who were diagnosed 2001-2018 (followed through 2019) and reported to have received initial chemotherapy for AML, SEER-17

				Relative survival (%) according to time since AML diagnosis			
				1-month	1-year	3-year	5-year
Subgroup/subtype	ICD-O-3 morphology/ behavior codes	N	Median age at diagnosis, years (IQR)	% (95% CI)	% (95% CI)	% (95% CI)	% (95% CI)
**APL (AML with t(15;17)(q22;q12))**	9866/3	**3,141**	**47 (35-59)**	**90.2 (89.1-91.2)**	**85.0 (83.7-86.2)**	**82.1 (80.6-83.5)**	**79.9 (78.2-81.4)**
**CBF AML**	*	**1,085**	**49 (35-62)**	**95.3 (93.8-96.4)**	**76.3 (73.6-78.8)**	**58.7 (55.5-61.7)**	**54.4 (51.2-57.6)**
AML, t(8;21)(q22;q22)	9896/3	597	50 (35-63)	96.4 (94.5-97.6)	73.3 (69.5-76.7)	52.6 (48.3-56.8)	48.0 (43.6-52.2)
AML with inv(16)(p13.1q22) or t(16;16)(p13.1;q22)	9871/3	488	47 (36-60)	93.9 (91.4-95.7)	79.9 (76.0-83.3)	66.1 (61.4-70.3)	62.2 (57.3-66.7)
**AML with antecedent condition/therapy**	*	**1,983**	**67 (57-74)**	**92.6 (91.4-93.7)**	**45.6 (43.3-47.8)**	**19.4 (17.6-21.3)**	**15.5 (13.8-17.3)**
AML with myelodysplasia-related changes	9895/3	1,848	67 (57-75)	92.3 (91.0-93.5)	44.8 (42.5-47.1)	19.1 (17.2-21.0)	15.3 (13.6-17.2)
Therapy-related myeloid neoplasm	9920/3	52	62 (54-72)	92.5 (80.8-97.2)	52.7 (38.1-65.3)	29.8 (17.9-42.7)	26.3 (14.9-39.2)
Therapy-related MDS, NOS†	9987/3	28	58 (49-71)	100 –	65.2 (44.2-79.9)	18.6 (6.7-35.1)	11.4 (2.9-26.5)
Refractory anemia with excess blasts in transformation‡	9984/3	52	66 (53-73)	98.3 (85.9-99.8)	53.0 (38.4-65.7)	19.1 (9.4-31.4)	10.8 (4.0-21.6)
Myeloid leukemia associated with Down syndrome	9898/3	<25	22 (21-38)	–	–	–	–
**All other AML**	*	**22,264**	**62 (49-72)**	**89.3 (88.9- 89.7)**	**51.6 (51.0-52.3)**	**29.7 (29.1-30.4)**	**25.4 (24.8-26.0)**
AML, NOS	9861/3	14,223	63 (50-72)	89.4 (88.8-89.9)	51.2 (50.4-52.0)	29.0 (28.2-29.8)	24.5 (23.8-25.3)
Myeloid sarcoma	9930/3	197	53 (39-66)	98.6 (95.3-99.6)	57.4 (50.1-64.0)	44.0 (36.8-51.0)	38.4 (31.2-45.6)
Acute panmyelosis with myelofibrosis	9931/3	109	62 (54-72)	98.3 (92.6-99.6)	72.9 (63.2-80.5)	41.1 (31.3-50.5)	24.1 (15.8-33.5)
AML with minimal differentiation	9872/3	617	62 (48-72)	88.5 (85.6-90.8)	49.6 (45.5-53.5)	30.0 (26.3-33.8)	24.6 (21.1-28.4)
AML without maturation	9873/3	1,115	59 (45-69)	89.9 (88.0-91.5)	53.2 (50.2-56.1)	31.8 (29.0-34.7)	28.2 (25.4-31.0)
AML with maturation	9874/3	1,153	60 (48-71)	93.1 (91.5-94.5)	57.2 (54.3-60.1)	33.4 (30.6-36.2)	28.1 (25.4-30.9)
Acute myelomonocytic leukemia	9867/3	2,305	59 (47-70)	87.3 (85.9-88.6)	51.6 (49.5-53.7)	30.9 (28.9-32.8)	26.9 (25.0-28.9)
Acute monoblastic and monocytic leukemia	9891/3	1,739	59 (46-69)	85.4 (83.7-87.0)	50.5 (48.1-52.9)	29.9 (27.7-32.2)	27.0 (24.8-29.2)
Acute erythroid leukemia	9840/3	384	63 (52-71)	94.2 (91.3-96.1)	45.2 (40.1-50.2)	21.0 (17.0-25.4)	16.8 (13.0-21.0)
Acute megakaryoblastic leukemia	9910/3	92	57 (44-67)	89.3 (80.8-94.1)	27.5 (18.8-36.9)	13.1 (7.0-21.0)	11.8 (6.1-19.6)
Acute basophilic leukemia	9870/3	<25	64 (47-82)	–	–	–	–
AML with t(9;11)(p22;q23)	9897/3	222	53 (38-66)	92.9 (88.6-95.6)	58.6 (51.7-64.8)	32.3 (26.1-38.7)	27.8 (21.7-34.3)
AML (megakaryoblastic) with t(1;22)(p13;q13)	9911/3	<25	58 (43-69)	–	–	–	–
AML with t(6;9)(p23;q34)	9865/3	46	61 (45-66)	93.6 (81.1-97.9)	66.0 (50.2-77.9)	34.4 (20.2-49.1)	23.3 (10.4-39.1)
AML, inv(3)(q21;q26.2) or t(3;3)(q21;q26.2)	9869/3	35	60 (42-70)	97.3 (80.7-99.6)	36.7 (20.9-52.5)	23.1 (10.2-38.9)	23.1 (10.2-38.9)

Abbreviations: AML, acute myeloid leukemia; APL, acute promyelocytic leukemia; CBF, core binding factor; CI, confidence interval; ICD-O-3, International Classification of Diseases for Oncology (third edition); IQR, interquartile range; MDS, myelodysplastic syndrome; NOS, noth otherwise specified; SEER-17, 17 cancer registry areas of the Surveillance, Epidemiology, and End Results Program; <25, data points with fewer than 25 cases are suppressed to protect patient confidentiality; –, not applicable.

* Includes ICD-O-3 codes for subtypes listed below the respective subgroup.

† Code became obsolete in 2010 and entity coded as 9920/3.

‡ Code obsolete in 2010 and entity coded as 9983/3.
